# Nursing Assessment of Pressure Injury Risk with the Braden Scale Validated against Sensor-Based Measurement of Movement

**DOI:** 10.3390/healthcare10112330

**Published:** 2022-11-21

**Authors:** Susan M. Kennerly, Phoebe D. Sharkey, Susan D. Horn, Jenny Alderden, Tracey L. Yap

**Affiliations:** 1 College of Nursing, East Carolina University, Greenville, NC 27858, USA; 2 School of Business, Loyola University Maryland Sellinger, Baltimore, MD 21210, USA; 3 School of Medicine, University of Utah, Salt Lake City, UT 84132, USA; 4 School of Nursing, Boise State University, Boise, ID 83702, USA; 5 School of Nursing, Duke University, Durham, NC 27710, USA

**Keywords:** pressure ulcer, pressure injury, Braden scale, older adults, movement, accelerometer, risk assessment, assessment accuracy, prevention

## Abstract

Nursing staff assessment to accurately identify pressure injury (PrI) risk is a hallmark in PrI prevention care. Risk scores from the Braden Scale for Predicting Pressure Sore Risk^©^ (hereafter Braden), a commonly used tool for assessing PrI risk, signal the need for preventative care. Braden Mobility, Activity, and Sensory Perception subscale subgroups associated with repositioning movement features help identify preventative strategies that minimize pressure intensity and duration. Evidence confirming subscale rating accuracy is needed. This study compared assessment score accuracy with movement data collected via accelerometer sensor. Sample included 913 nursing home residents from the Turn Everyone and Move for Pressure Ulcer Prevention (TEAM-UP) cluster randomized trial. Movements and Braden Mobility and Activity subscale scores were evaluated for significant differences and associations. Mobility subgroups explained a small-medium amount of variance in mean lying and upright movement features (0.002 ≤ *R*^2^ ≤ 0.195). Activity subgroups explained a small-medium amount of variance in mean lying, upright, and ambulating movements (0.016 ≤ *R*^2^ ≤ 0.248). Significant associations occurred among subscale subgroups and most movements. Nursing assessment ratings using Braden scale’s Mobility and Activity subscale scores are accurate indicators of actual repositioning movements and can be relied upon for PrI prevention care planning for older adults.

## 1. Introduction

Pressure injury (PrI), damage to skin and underlying tissues associated with prolonged pressure, is an enormous and costly problem in healthcare settings [1] and is largely preventable [2]. External pressure exposes an area of tissue to high levels of constant pressure that if unrelieved by repositioning movement prevents tissue reperfusion and places the resident at risk for PrI development. Nursing assessment is pivotal to determine whether a nursing home (NH) resident is at risk for development of a PrI and requires establishment of an effective preventive care plan. The goal in assessment of NH residents is to accurately identify an individual resident’s status regarding a set of internationally accepted factors considered to predispose skin and tissues to develop PrI. The risk assessment result denotes what level of risk an individual has for PrI development and serves as a trigger for nurses to initiate appropriate prevention strategies to deter PrI occurrence [3]. Repositioning, a primary preventive strategy used to minimize the intensity and duration of pressure, can reduce the likelihood of developing a PrI. This study investigated the correspondence between repositioning movements of NH residents that were measured using a triaxial accelerometer and nursing estimates of PrI risk using the Mobility and Activity subscale scores of the Braden Scale for Predicting Pressure Sore Risk^©^ (hereafter, Braden) [3,4].

The Braden Scale [3,4], the most frequently used PrI risk assessment tool in the United States, was developed for use in NHs and has demonstrated reliability and validity [5,6]; in fact, its sensitivity and specificity is unmatched when compared to Norton and Waterlow assessment scales [7]. A nurse’s estimate of overall risk (Braden Total risk score) relies upon clinical judgment and is dependent on the accuracy of ratings assigned to each of six Braden Scale subscales (Mobility, Activity, Sensory Perception, Nutrition, Friction/Shear, Moisture); each subscale has been established as valid [3,5,8,9] and can be used independently. Numerous studies have been conducted further validating the tool since its initial development, but most have focused on the predictive validity of the overall risk (Braden Total risk score) relative to PrI development [10] rather than whether the score gives an accurate indication of PrI risk. Overall risk often serves to signal the need for using preventative protocols, but it is the individual subscale, such as Mobility subscale or Activity subscale, that provides insights into the design of specific interventions aimed at preventing the PrI [11]. Intervention guides most often associate repositioning and movement as essential components of preventative strategies to minimize intensity and duration of pressure and rely upon the Braden Scale’s Mobility, Activity, and Sensory Perception subscale ratings to establish the plan of care [12,13]. Evidence is needed to confirm the accuracy of nursing assessment subscale ratings and clarify their conceptual linkages.

**Figure 1:**
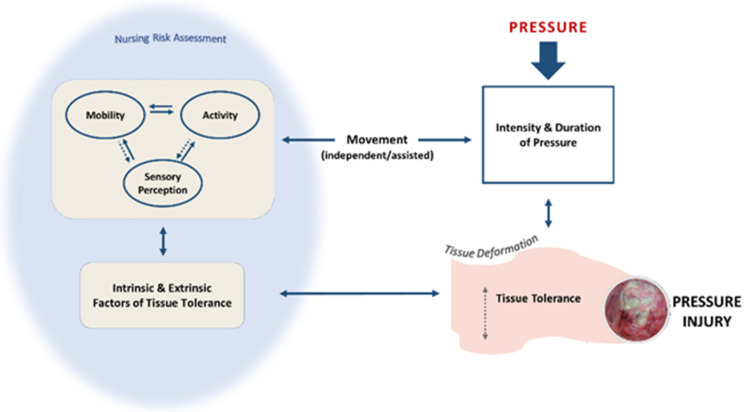
Influence of Mobility, Activity, and Sensory Perception Risk Assessment Dimensions and Movement on Pressure Intensity and Duration and Pressure Injury.

Braden and Bergstrom framed the conceptual foundation for understanding overall risk of PrI (historically called decubiti, pressure sores, or pressure ulcers) and the etiology of its development as based on two areas: intensity and duration of pressure and tissue tolerance [14,15]. Figure 1 depicts relationships between these two areas and the dimensions influencing each. The first conceptual area, intensity and duration of pressure, is thought to be influenced by mobility, activity, and/or sensory perception dimensions. Mobility and activity are defined, respectively, as movement in bed or movement out-of-bed and are hypothesized to directly influence each other based on repositioning movement pattern. Mobility and activity dimensions affect the overall contribution movement makes to avoiding, removing, or reducing pressure exposure. Sensory perception is defined as the “ability to perceive or respond to discomfort by changing position or requesting assistance to change position” [3] (p. 9). Intact sensory perception exerts an indirect influence on repositioning movement by first sensing pressure and, then, cueing an individual to change body position through mobility and activity. Thus, sensory perception is a dimension commonly understood to impact the degree to which mobility and activity are performed. All three dimensions, mobility, activity, and sensory perception, are often targeted for nursing care interventions aimed at managing pressure exposure [11]. The second conceptual area, tissue tolerance, refers to the ability of skin and underlying tissues to withstand pressure and subsequent damage. Extrinsic (moisture, presence of friction and shear) and Intrinsic (e.g., nutritional status, age) dimensions affecting tissue tolerance determine how long and how much pressure an individual can withstand without tissue damage occurring. Repositioning movement like that Braden and Bergstrom describe as occurring in mobility and activity can minimize pressure intensity and duration and reduce the likelihood of PrI development. The dynamic interaction between repositioning movement, pressure intensity and duration, and tissue tolerance plays a critical role in determining whether PrI will develop. This study examines the accuracy of nursing assessment using the Braden Mobility and Activity subscales to estimate PrI risk associated with repositioning movements and the potential influence of varied levels of sensory perception.

## 2. Methods

### 2.1. Design, Sample, and Setting

A retrospective design was used to examine nursing assessment accuracy of Braden Mobility and Activity subscales by comparing subscale assessment scores to triaxial accelerometer movement data. A wearable resident specific triaxial accelerometer sensor worn on the upper chest was part of a wireless monitoring system that tracked movements associated with body position change while upright (sitting in bed or chair) or lying (in bed or reclining chair) and ambulating. Movements were documented every 10 s, 24 h a day. These movement data were selected as empirical indicators of repositioning and were compared to Braden Mobility and Activity subscale scores because these subscales are defined according to levels of resident ability/limitation with regard to changing position in bed (Mobility) and out-of-bed and ambulating (Activity). Analyses compared NH resident movement data and nursing staff Braden PrI risk assessments collected during the cluster randomized trial 1R01NR016001 Turn Everyone and Move for Pressure Ulcer Prevention (TEAM-UP) conducted from 2016–2021 in nine U.S NHs that were Medicare and Medicaid certified skilled nursing facilities [16]. Repositioning movement data from the TEAM-UP trial were collected from NHs randomly assigned a NH-wide 2-, 3-, or 4 h repositioning interval as standard of care for the duration of the trial’s 4-week intervention period.

Secondary data examined in the current study were comprised of N = 913 Team-UP trial residents who had at least one day with a complete record of 22 to 24 h of triaxial accelerometer movement data during the trial’s intervention period and whose initial Braden assessment included Mobility and Activity subscale scores. Study variables described in detail in the Measures section below included repositioning movement data (upright, lying, ambulating) from triaxial accelerometers and residents’ demographics (age, gender, race, ethnicity, and diagnoses) and nursing staff Braden Scale Total score and subscale risk assessment scores extracted from the electronic health record.

### 2.2. Measures

#### 2.2.1. Braden Scale and Subscales

Braden Scale Total score and subscale scores were obtained from the initial Braden weekly assessment documented in the electronic health record for each resident during the TEAM-UP trial intervention period. Risk assessment for each resident was completed by nursing staff who were trained by the NH company in use of the Braden Scale and were familiar with each resident’s physical and functional abilities. The Braden Scale is comprised of six discrete subscales (Sensory Perception, Activity, Mobility, Moisture, Nutrition, and Friction and Shear) with five of the six scored on a scale of 1–4 progressing from most to least severity of alteration; Friction and Shear scores range from 1–3 [3]. Overall PrI risk is reflected by the Braden Scale Total score ranging from 6 to 23 that results when all six subscale scores are summed. The lower the total score the greater the estimated risk of developing a PrI. Risk scores can be categorized as low (19–23), mild (15–18), moderate (13–14, high (10–12), and severe (≤9) risk; residents with severe risk were not eligible for inclusion in the TEAM-UP trial. Mobility, Activity, and Sensory Perception subscales defined below [3] are the Braden subscales of primary interest in this study since these scores reflect an individual’s ability to respond to pressure on skin and tissues.

- Mobility subscale—“ability to change and control body position” (p. 8), moving while lying or reclining in bed or chair;
- Activity subscale—ability to release pressure from or “avoid intense and prolonged pressure over vulnerable skin areas” (p. 8) and indicates how much or how little the resident moves independently while out of bed or in a wheelchair;
- Sensory Perception subscale—“ability to perceive or respond to discomfort by changing position or requesting assistance to change position” (p. 9)

Mobility and Activity subscales describe the ability of a resident to bring about a change in body position and would exert a direct influence on the frequency and duration of repositioning movements. In contrast, the Sensory Perception subscale describes a dimension that may indirectly affect whether a resident initiates or requests assistance with repositioning movement.

#### 2.2.2. Repositioning Movement

Repositioning movement included frequency of change in and duration of body position orientation when a resident is either in or out-of-bed, including ambulating for all residents with at least one day with a complete record of 22–24 h of movement data. Movement data for partial days were not included in the analyses. Frequency and duration of three movement features (Upright, Lying, and Ambulating) were extracted from triaxial accelerometer data collected every 10 s, 24 h a day via a personal sensor worn on the anterior chest by each resident during the TEAM-UP intervention period [16]. Even though a NH-wide 2-, 3-, or 4 h repositioning interval was assigned during TEAM-UP, resident rights were retained to refuse repositioning care or to move more often. Movement frequency was the number of times per day a resident changed position. For example, a single change from previous upright position to lying position would be recorded as a one movement change to a lying position. Duration was the number of minutes per day spent in a position. For example, a resident who maintains a lying position for at least 15 min or more would be recorded as remaining in the lying position for the actual number of minutes. Frequencies and durations were accrued daily and separately for each resident’s upright, lying, and ambulating position(s). The triaxial accelerometer measured acceleration along forward, back, and left/right axes according to the sensor’s detection of the respective body position orientation maintained for at least 15 min. Detection of body position orientation was based on preset thresholds as listed below.

- Upright position—≥50 degrees upright angle of resident’s torso to ground when standing or sitting in bed or chair. Tilt angle ≥ 10 degrees signified a hip-to-hip weight shift to right or left while upright.
- Lying position—<50 degrees upright angle of resident’s torso to ground when facing upward. Roll angle ≥20 degrees signified a left or right change in position while lying.
- Ambulating position—≥9 steps of forward movement within the resident’s room or other location at NH.

Upright and Lying movement features were both representative of Mobility subscale scores 1–4 and Activity subscale scores 1–4. Ambulating movement features were only representative of Activity subscale scores 3–4 that are, respectively labeled by Braden and Bergstrom as “walks occasionally” and “walks frequently” [3].

### 2.3. Statistical Analysis

Statistical analyses were conducted using Statistical Analysis System (SAS version 9.4) [17] software and included descriptive statistics (means, standard deviations, frequencies, percentages) to summarize demographic attributes, Braden Total, Mobility, Activity, and Sensory Perception subscale scores, and movement feature characteristics. The analyzed sample contained no missing data. Movement features and Braden subscale scores were evaluated for significant differences using analysis of variance (ANOVA) and multiple pairwise comparison scores (Tukey–Kramer method with unequal sample size subgroups). Bivariate Pearson correlations were calculated to see how highly correlated the Braden Mobility, Activity, and Sensory Perception subscale scores were with each other and with movement features. Multiple ordinary least squares regression was used to determine the contribution of Braden subscale scores represented as binary dummy variable constructs in predicting movement feature outcome variables.

## 3. Results

Data were received from nine study NHs that ranged in size from 126–238 operating beds and were Medicare and Medicaid certified skilled nursing facilities located in five states within central and eastern U.S. regions. Table 1 presents demographic characteristics for the sample (N = 913). Average age was 77.7 years (SD = 13.1). Most were of White race (65.5%), female gender (61.8%), and not of Hispanic or Latino ethnicity (97.7%). More than 80% of the residents had difficulty walking or muscle weakness/wasting. Overall, the sample’s average risk for PrIs was mild (Braden Scale Total score mean = 17.5, SD = 3.0). Residents were found to have fewer challenges with Sensory Perception (mean = 3.7, SD = 0.6) than with either Mobility (mean = 3.0, SD = 0.8) or Activity (mean = 2.5, SD = 0.8). The daily movement data were based on a varied number of days of observations (1–28 days, mean = 16 days) for each resident. Movement features confirmed that residents spent more time lying in bed or reclining in a chair with few body position changes. In fact, residents on average spent about 15 h in a lying position compared to 8 h upright in bed or chair. Residents who ambulated did so for short time periods, about 3.5 min, and on average just over 7 times a day.

### 3.1. Mobility and Activity Subscale Results

Table 2 and Table 3 compare movement features by subgroups defined by the Braden Scale Mobility and Activity subscale scores (1–4), respectively. Overall, variation in the residents’ accelerometer measurements aligned with Mobility and Activity subscale score ratings describing different movement patterns among the subgroups.

#### 3.1.1. Mobility Subscale Results

Mobility subscale scores collectively explained a small to medium amount of the variance [18] in mean lying and upright movement features (0.002 ≤ *R*^2^ ≤ 0.195) for the NH residents (Untabled). Significant mean movement feature differences were found among most of the Mobility subscale subgroups, except for mean lying frequency/day where no pairwise significant differences were found (Table 2). The magnitude of change in lying duration/day significantly decreased (*p* < 0.001) as Mobility subscale scores increased; also, almost all pairwise subgroup comparisons differed (*p* < 0.05). Lying frequency/day was not significantly different (*p* = 0.568) across Mobility subscale subgroups. In contrast, the magnitude of upright duration/day and frequency/day significantly increased (*p* < 0.001) as Mobility subscale scores increased. Ambulating duration and frequency features are not included in Table 2; residents assessed for Mobility are not judged by walking and out-of-bed movements that are part of the Activity subscale dimensions.

#### 3.1.2. Activity Subscale Results

Activity subscale scores collectively explained a small to medium amount of the variance [18] in mean lying, upright, and ambulating movement features (0.016 ≤ *R*^2^ ≤ 0.248) (Untabled). Generally, how long and how much movement occurred differed significantly (*p* < 0.001) among residents classified as either Activity1 (bedfast), Activity2 (chairfast), Activity3 (walking occasionally), and Activity4 (walking frequently). Pairwise comparisons of mean movement differences between residents rated as Activity 1 compared to all other Activity subscale subgroups were statistically significant (*p* < 0.05) in distinguishing variations in lying and upright movements. Pairwise mean movement feature differences between residents with an Activity3 score and residents with an Activity4 score were only significant when comparing ambulating durations and frequencies.

Like the Mobility subscale, pairwise mean differences in movement features were generally largest between residents in the lowest and highest Activity subscale subgroups (Activity1 to Activity4). However, there was one exception to this trend. Upright duration (min)/day pairwise mean differences were lower between Activity1 and Activity4 subgroup comparisons [mean difference = 377.5] than between Activity 1 and Activity 3 score group comparisons [mean difference = 395.0]; i.e., residents who were frequent walkers spent less time up in chairs or in bed than infrequent walkers.

### 3.2. Mobility, Activity, and Sensory Perception Subscale Association Results

Bivariate Pearson-product-moment correlations (Table 4) were estimated to determine the extent to which the NH resident Braden Scale Mobility, Activity, and Sensory Perception subscales and accelerometer system movement feature measures were associated and to better understand how these measures are related. Significant associations were demonstrated among the Braden subscales and most movement features. Mobility and Activity subscales had large correlation [r = 0.64, *p* < 0.001] denoting that resident mobility and activity measures increased or decreased together. The Sensory Perception subscale had a smaller, medium correlation with the Mobility subscale [r = 0.44, *p* < 0.001] and Activity subscale [r = 0.33, *p* < 0.001]. All three Braden subscales were positively associated with upright and ambulating movement feature measures and negatively associated with lying duration/day, though only Mobility and Sensory Perception were not significantly related to lying frequency/day of residents as demonstrated in the ANOVA tests.

Ordinary least squares regression analyses (Table 5) were used to determine the extent to which resident movement feature measures could be predicted by Braden Scale Mobility and Activity subscale constructs and examine the influence of Sensory Perception subscale constructs. The binary dummy variables representing Mobility and Activity subscale scores collectively and individually explained variation in resident movement features. Changes in movement values generally followed logical progressions with changes in Mobility and Activity subscale score values, moving in the same direction if the measures were directly related or opposite directions if there was an inverse relationship. Magnitude of change in movement features (outcome variables) was evaluated for each subscale level 1–3 relative to the respective Braden subscale level 4 reference group (Mobility 4, no limitations; Activity 4, walks frequently; Sensory Perception 4, no impairment).

Ambulating Duration and Frequency movement feature measures were not evaluated for the Mobility subscale since it refers to in-bed movement. Activity subscale scores 1 (bedfast), 2 (chairfast), and 3 (walks occasionally) were significant (*p* < 0.001) predictors of Total Ambulating Duration (min/day) and Total Ambulating Frequency/day. The addition of Braden Sensory Perception subscale scores as independent predictor variables did not produce any change in the significant main effects for Mobility, Activity, or movement outcomes. The only significant additional contribution of Sensory Perception was in Mean Upright Frequency and Total Ambulating Duration per day.

## 4. Discussion

International guidelines emphasize formally assessing PrI risk factors affecting skin and tissue tolerance [2]. The goal in risk assessment is to accurately identify an individual’s overall risk. Nursing staff have executed comprehensive PrI risk assessments for over 50 years by using a variety of assessment tools. The Braden Scale, developed for formal assessment in the late 1980′s, rapidly became the most commonly used tool for assessing PrI risk in most United States healthcare settings. Braden and Bergstrom [3] envisioned that risk assessment would trigger use of appropriate prevention strategies to avert PrI development, and, indeed, that has occurred. However, studies examining whether the tool accurately predicts PrI occurrence have dominated research efforts, even though the tool’s stated aim is to prevent PrI development [19]. Furthermore, questions clinicians raise about the tool’s accuracy in reflecting risk have gone unanswered. This study addresses the need for clarification about accuracy of the tool in estimating risk rather than injury development in a current healthcare environment. The study demonstrates that licensed nursing staff assessment ratings using the Braden Scale’s Mobility and Activity subscale scores are accurate indicators of actual repositioning movements and can be relied upon for PrI prevention care planning for older adults.

Definitions that Braden, Bergstrom, and colleagues [3] provided for level 1–4 risk (high to low risk) dimensions of Mobility (in-bed) and Activity (out-of-bed) offer unique representations about expected movement. Nursing staff were found to have accurately interpreted the levels for these dimensions among the individuals studied as evidenced by significant differences among subscale subgroups that distinguished lying, upright, or ambulating movements.

Mobility and Activity subscale score distributions included all 4 risk levels that enabled a thorough evaluation of the risk assessment ratings and rendered a high level of confidence in the correspondence found between nursing assessment ratings and actual movements. Similar patterns of movement were found across Mobility and Activity for risk level ratings. For example, an individual confined to bed (Activity 1) or completely immobile (Mobility 1) would be expected to engage in longer durations in a lying position; and, as described in the results, a statistically significant decrease in lying duration was found as Mobility and Activity risk level ratings increased to levels 3–4 in which the individual had slight to no limitation in mobility or was out-of-bed walking.

A primary distinguishing feature of Activity versus Mobility subscale dimensions is presence of ambulating movements (Activity 3, walks occasionally; Activity 4, walks frequently). This clinical distinction explains the low amount of ambulating movement detected for those rated as Activity 1 or 2 who spend much time in bed or chair and, similarly, the decreased lying duration for those rated as Activity 3 or 4. Mobility levels of individual residents were more likely distinguished by the number of times an upright position was assumed and duration spent in lying and upright positions than the number of times residents lie in bed or chair during a day. Repositioning movements observed in this study affirm the conceptualized levels of movement associated with risk assessment according to Braden Scale Mobility and Activity subscales, i.e., the higher the subscale score the lower the PrI risk and the higher the movement feature values that reflect a more mobile or more active individual.

Numerous upright, lying, and ambulating movements observed in this study are consistent with those typically expected to achieve, prevent, or offload pressure that may contribute to PrI development. Exploration of the conceptualized dynamic relationship between mobility, activity, and sensory perception yielded mixed findings. Overall, small to medium bivariate correlations resulted between movement features with both Mobility and Activity subscale scores, whereas correlations with Sensory Perception subscale scores were small. These findings suggest that while mobility, activity, sensory perception, and movement are related concepts, the extent to which individuals are mobile and active is more directly connected to movement than the ability to feel and communicate discomfort. Braden Sensory Perception subscale scores did not change the strength or direction of relationships between Braden Mobility and Activity subscale scores and movement features. While these subscale measures were significantly related and moved together in the same direction, correlation of Sensory Perception was not strong with either Mobility or Activity using Braden Scale assessment. This could have been because the mean Sensory Perception score was 3.7 indicating most residents had little to no impairment. Presence of an association between sensory perception and movement features may be stronger with a sample that includes more older adults experiencing severe limitations (Sensory Perception 1 = completely limited) in sensory perception.

Mobility, activity, and sensory perception are the three dimensions of overall risk that are often targeted for nursing care interventions aimed at managing pressure exposure [10]. Yet, whether an individual is deemed to be at-risk for PrI development is driven by the Braden Scale Total score that is intended to reflect overall PrI risk. In recent years, there has been growing critique of the use of the Braden Scale score as the primary indicator of risk even though evidence about its sensitivity and specificity show the Braden Scale’s validation is unmatched when compared to Norton and Waterlow assessment scales [7]. Kottner [7] noted that there is no universal standard point of reference available that quantifies risk. These concerns about the Braden and risk assessment in general and the ongoing high PrI incidence rates for in-patient care stays in NHs and hospitals have prompted some to suggest that an augmented or additional means of risk determination is needed [20] and others to propose the addition of more dimensions to the Braden Scale to better explain risk [21]. In contrast, findings from this study strengthen confidence in the accuracy of Mobility and Activity subscale risk dimension scores, support their clinical value as a guide for prevention care, and echo the importance that international PrI prevention and treatment guidelines [2] attribute to repositioning movement as an essential part of minimizing the intensity and duration of pressure. Additionally, study findings suggest that Mobility and Activity subscales can make a meaningful contribution to prediction of PrI risk. Consideration should be given to rethinking the current practice of defining risk as the sum of all six risk dimensions versus use of a more targeted approach. Using subscale scores for dimensions such as Mobility and/or Activity to distinguish or further illuminate risk could potentially offer better insights into PrI prevention care planning than solitary use of an overall threshold risk score to trigger that planning.

### Limitations

This study had several limitations that may affect generalizability despite use of rigorous methods to ensure appropriateness of study design and analyses. Analyses of movement data were confined to examination of individuals having a complete record of 22 to 24 h for one or more days. Measuring movement data daily required removal of data for partial days (<22 h/day) and possibly affected the mean duration and frequency of the movements studied.

The sample studied relatively small numbers of participants in some Braden subscale categories. For example, the relatively small sample size in some Mobility levels may have limited ability to detect significant differences between a few level comparisons, e.g., level 1 and 2 ratings. Additionally, the presence of only 1 resident with a Sensory Perception level 1 score could have affected the detection of an influence on movement and on Mobility and Activity subscale scores.

The older adults in this study were assessed for PrI risk according to standardized guidance for conducting assessments with the Braden Scale, but these individuals may have experienced health conditions that differ from other clinical settings. However, the focus of the study was on correspondence of nursing staff risk assessment ratings with actual movement data. There is no evidence that nursing staff assessment ratings would not be accurate when applied to older adults in other care settings.

## Figures and Tables

**Table 1:** Demographic, Braden Scale, and Movement Feature Characteristics (N = 913).

Characteristic	Total Sample
Age in Years mean (SD)	77.7 (13.1)
Age Distribution N (%)	
≤64 years	153 (16.8)
65–70 years	110 (12.1)
71–80 years	216 (23.6)
81–85 years	126 (13.8)
86–89 years	115 (12.6)
≥90 years	193 (21.1)
Gender: Male N (%)	349 (38.2)
Race ^∬^ N (%)	
Black	260 (28.5)
White	598 (65.5)
Other	55 (6.0)
Ethnicity N (%)	
Hispanic or Latino	21 (2.3)
Not Hispanic or Latino	892 (97.7)
Top Diagnoses N (%)	
Difficulty walking	779 (85.3)
Muscle weakness/wasting	747 (81.8)
Difficulty with swallowing or speech	502 (55.0)
Hypertension	429 (47.0
Atherosclerotic heart disease	280 (30.7)
Alzheimer’s disease & related dementias	450 (49.3)
Gastroesophageal reflux disease (GERD)	273 (29.9)
Depression	213 (23.3)
Diabetes, Type 2	197 (21.6)
Cerebrovascular disease	190 (20.8)
Braden Scale Scores mean (SD)	
Mobility	3.0 (0.8)
Activity	2.5 (0.8)
Sensory Perception	3.7 (0.6)
Total Braden Score	17.5 (3.0)
Movement Features mean (SD)	
MEAN Lying Duration (min)/Day	897.9 (264.8)
MEAN Lying Frequency/Day	105.6 (87.3)
MEAN Upright Duration (min)/Day	477.6 (261.3)
MEAN Upright Frequency/Day	221.0 (175.0)
Total Ambulating Duration (min)/Day	25.5 (51.6)
Total Ambulating Frequency/Day	7.3 (11.2)

^∬^ % American Indian/Alaska Native = 0. % More than one race = 0. % Native Hawaiian or Other Pacific Islander = 0.

**Table 2:** Examining Mean Differences Between Braden Mobility Subscale Score Groups Relative to Movement Features (N = 913).

	Braden Mobility Subscale Score ^§^				Pairwise Comparisons		ANOVA	
Movement Features	1 (n = 28)	2 (n = 236)	3 (n = 349)	4 (n = 300)	Subgroups	Mean Difference	*F*	*p*
MEAN Lying Duration	1149.4	1024.5	874.6	801.8	1–2	124.9	46.56	<0.001
(min)/Day mean (SD)	(276.2)	(270.7)	(242.8)	(228.7)	1–3	274.8 *		
					1–4	347.7 *		
					2–3	149.9 *		
					2–4	222.8 *		
					3–4	72.9 *		
MEAN Lying	116.0	100.0	109.4	104.5	1–2	16.0	0.67	0.568
Frequency/Day mean (SD)	(127.9)	(73.0)	(95.2)	(85.2)	1–3	6.6		
					1–4	11.5		
					2–3	9.3		
					2–4	4.5		
					3–4	4.8		
MEAN Upright Duration	236.7	361.5	507.8	556.2	1–2	124.9	38.22	<0.001
(min)/Day mean (SD)	(281.7)	(269.2)	(242.6)	(228.6)	1–3	271.1 *		
					1–4	319.5 *		
					2–3	146.2 *		
					2–4	194.6 *		
					3–4	48.4		
MEAN Upright	69.6	117.6	228.8	307.5	1–2	48.0	73.2	<0.001
Frequency/Day mean (SD)	(96.9)	(135.7)	(166.2)	(166.5)	1–3	159.2 *		
					1–4	238.0 *		
					2–3	111.2 *		
					2–4	190.0 *		
					3–4	78.8 *		

^§^ Four population subgroups based on residents’ Braden Mobility Subscale Score 1, 2, 3, or 4 with sample size for each subgroup (n = xxx). * Statistically significant *p* ≤ 0.05 using Tukey–Kramer method for pairwise comparisons with unequal sample size subgroups.

**Table 3:** Examining Mean Differences Between Braden Activity Subscale Score Groups Relative to Movement Features (N = 913).

	Braden Activity Subscale Score ^§^				Pairwise Comparisons		ANOVA	
Movement Features	1 (n = 58)	2 (n = 463)	3 (n = 259)	4 (n = 133)	Subgroups	Mean Difference	*F*	*p*
MEAN Lying Duration	1219.9	934.4	816.7	788.3	1–2	285.5 *	55.75	<0.001
(min)/Day mean (SD)	(192.1)	(265.0)	(233.6)	(202.0)	1–3	403.2 *		
					1–4	431.6 *		
					2–3	117.7 *		
					2–4	146.1 *		
					3–4	28.4		
MEAN Lying	146.1	105.3	102.4	94.9	1–2	40.8 *	4.94	0.002
Frequency/Day mean (SD)	(115.8)	(88.4)	(87.9)	(64.6)	1–3	43.7 *		
					1–4	51.2 *		
					2–3	2.9		
					2–4	10.4		
					3–4	7.5		
MEAN Upright Duration	166.7	450.3	561.7	544.2	1–2	283.5 *	47.08	<0.001
(min)/Day mean (SD)	(191.8)	(263.2)	(235.0)	(204.6)	1–3	395.0 *		
					1–4	377.5 *		
					2–3	111.4 *		
					2–4	94.0 *		
					3–4	17.5		
MEAN Upright	58.2	178.6	283.1	318.9	1–2	120.4 *	60.40	<0.001
Frequency/Day mean (SD)	(117.7)	(164.6)	(166.1)	(147.1)	1–3	224.8 *		
					1–4	260.7 *		
					2–3	104.5 *		
					2–4	140.3 *		
					3–4	35.8		
TOTAL Ambulating Duration	1.0	11.4	32.2	72.4	1–2	10.5	65.23	<0.001
(min)/Day mean (SD)	(3.9)	(38.1)	(42.1)	(81.2)	1–3	31.2 *		
					1–4	71.4 *		
					2–3	20.7 *		
					2–4	60.9 *		
					3–4	40.2 *		
TOTAL Ambulating	0.4	3.3	10.2	18.3	1–2	2.9	99.98	<0.001
Frequency/Day mean (SD)	(1.3)	(8.1)	(8.6)	(16.5)	1–3	9.8 *		
					1–4	17.9 *		
					2–3	6.8 *		
					2–4	14.9 *		
					3–4	8.1 *		

**^§^** Four population subgroups based on residents’ Braden Activity Subscale Score 1, 2, 3 or 4 with sample size for each subgroup (n = xxx). * Statistically significant *p* ≤ 0.05 using Tukey–Kramer method for pairwise comparisons with unequal sample size subgroups.

**Table 4:** Pearson Correlations of the Braden Scale Subscale Scores and Movement Features (N = 913).

		1	2	3	4	5	6	7	8	9
	Variable	Mobility Subscale	Activity Subscale	Sensory Perception	Lying Duration	Lying Frequency	Upright Duration	Upright Frequency	Ambulating Duration	Ambulating Frequency
**1**	Mobility Subscale	1.00								
**2**	Activity Subscale	0.64 ***	1.00							
**3**	Sensory Perception Subscale	0.44 ***	0.33 ***	1.00						
**4**	MEAN Lying Duration (min)/Day	−0.36 ***	−0.35 ***	−0.20 ***	1.00					
**5**	MEAN Lying Frequency/Day	0.01	−0.09 **	−0.02	0.26 ***	1.00				
**6**	MEAN Upright Duration (min)/Day	0.32 ***	0.30 ***	0.18 ***	−0.98 ***	−0.23 ***	1.00			
**7**	MEAN Upright Frequency/Day	0.44 ***	0.39 ***	0.27 ***	−0.68 ***	0.12 ***	0.67 ***	1.00		
**8**	TOTAL Ambulating Duration (min)/Day	0.32 ***	0.41 ***	0.20 ***	−0.29 ***	−0.06 *	0.14 ***	0.28 ***	1.00	
**9**	TOTAL Ambulating Frequency/Day	0.40 ***	0.49 ***	0.23 ***	−0.37 ***	−0.06	0.24 ***	0.39 ***	0.87 ***	1.00

* 0.011 ≤ *p* ≤ 0.050, ** 0.001 ≤ *p* ≤ 0.01, *** *p* < 0.001.

**Table 5:** Ordinary Least Squares Regression Models Predicting Movement Features Using Dummy Variable Constructs for Each Braden Mobility, Activity, and Sensory Perception Subscale Score (N = 913).

Movement Feature	Braden Subscale *	B	*p*	*R* ^2^
MEAN Lying Duration (min)/Day	Mobility1	191.23	<0.001	0.189
	Mobility2	132.97	<0.001	
	Mobility3	39.34	0.076	
	Activity1	302.56	<0.001	
	Activity2	64.48	0.029	
	Activity3	6.62	0.808	
	Sensory Perception2	37.49	0.340	
	Sensory Perception3	15.91	0.427	
MEAN Lying Frequency/Day	Mobility1	−23.42	0.227	0.025
	Mobility2	−26.24	0.010	
	Mobility3	−5.36	0.507	
	Activity1	69.85	<0.001	
	Activity2	22.52	0.036	
	Activity3	10.22	0.303	
	Sensory Perception2	6.86	0.631	
	Sensory Perception3	2.46	0.736	
MEAN Upright Duration	Mobility1	−184.41	<0.001	0.167
(min)/Day	Mobility2	−125.06	<0.001	
	Mobility3	−32.51	0.143	
	Activity1	−256.04	<0.001	
	Activity2	−18.88	0.523	
	Activity3	35.93	0.188	
	Sensory Perception2	−38.09	0.332	
	Sensory Perception3	−12.84	0.521	
Mean Upright Frequency/Day	Mobility1	−138.19	<0.001	0.231
	Mobility2	−121.95	<0.001	
	Mobility3	−48.92	<0.001	
	Activity1	−141.35	<0.001	
	Activity2	−57.48	0.003	
	Activity3	−9.64	0.583	
	Sensory Perception2	−62.47	0.014	
	Sensory Perception3	−24.06	0.062	
TOTAL Ambulating Duration (min)/Day	Activity1	−66.66	<0.001	0.183
	Activity2	−57.80	<0.001	
	Activity3	−39.27	<0.001	
	Sensory Perception2	−9.72	0.187	
	Sensory Perception3	−9.28	0.015	
TOTAL Ambulating Frequency/Day	Activity1	−16.94	<0.001	0.253
	Activity2	−14.30	<0.001	
	Activity3	−7.91	<0.001	
	Sensory Perception2	−2.73	0.073	
	Sensory Perception3	−1.53	0.053	

* Braden Subscale Dummy Variables for residents with Mobility 1, 2, or 3, Activity 1, 2, or 3, and Sensory Perception 2 or 3. Mobility 4, Activity 4, and Sensory Perception 4 were reference groups. There were no residents with a level 1 Sensory Perception subscale score.

## Data Availability

Data used in this publication include protected health information, and therefore cannot be freely shared. Data sharing will be possible with case-by-case approval from our institution’s Institutional Review Board; requests may be directed to the corresponding author.
